# Advanced Therapies for Inflammatory Bowel Disease and Risk of Skin Cancer: What’s New?

**DOI:** 10.3390/cancers17101710

**Published:** 2025-05-20

**Authors:** Sarah Bencardino, Francesca Bernardi, Mariangela Allocca, Alessandra Zilli, Federica Furfaro, Laurent Peyrin-Biroulet, Silvio Danese, Ferdinando D’Amico

**Affiliations:** 1 Gastroenterology and Endoscopy, IRCCS Ospedale San Raffaele, Vita-Salute San Raffaele University, 20132 Milan, Italy; bencardino.sarah@hsr.it (S.B.); bernardi.francesca@hsr.it (F.B.); allocca.mariangela@hsr.it (M.A.); zilli.alessandra@hsr.it (A.Z.); furfaro.federica@hsr.it (F.F.); danese.silvio@hsr.it (S.D.); 2 Department of Gastroenterology, INFINY Institute, INSERM NGERE, CHRU Nancy, F-54500 Vandœuvre-lès-Nancy, France; l.peyrin-biroulet@chru-nancy.fr

**Keywords:** skin cancer, inflammatory bowel disease, personalized medicine

## Abstract

**Introduction:** The introduction of biologic therapies and small molecule drugs has revolutionized the management of inflammatory bowel disease (IBD), providing targeted control of inflammation. However, concerns remain regarding their long-term safety profiles, particularly in relation to cancer risk. Chronic inflammation and immunosuppressive therapies contribute to malignancy risk, including skin cancers, such as melanoma and non-melanoma skin cancer (NMSC). This review examines the evidence on skin cancer risks associated with these therapies, focusing on specific drug classes and their mechanisms. **Results:** Tumor necrosis factor (TNF) inhibitors have shown conflicting evidence regarding melanoma risk, with some studies reporting a modest increase and others finding no significant association. Anti-integrin agents, such as vedolizumab, and interleukin (IL)-12/23 inhibitors, including ustekinumab, have demonstrated favorable safety profiles with minimal skin cancer risks. Selective IL-23 inhibitors and sphingosine-1-phosphate (S1P) receptor modulators have limited long-term data, but early findings indicate a low incidence of skin malignancies. Janus kinase (JAK) inhibitors do not show an increased risk of skin cancers in IBD. **Conclusions**: Current evidence suggests that skin cancer risk in IBD patients treated with biologics and small molecule drugs varies by drug class. TNF inhibitors and JAK inhibitors are associated with higher risks, while other therapies show lower malignancy risks. Regular skin cancer screening and protective measures remain critical, particularly for patients with additional risk factors. Further long-term studies are essential to refine safety profiles and inform clinical practice in this evolving therapeutic landscape.

## Simple Summary

The use of biologic therapies and small molecule drugs has significantly improved the treatment of inflammatory bowel disease by targeting inflammation more effectively. However, there are concerns about their long-term safety, particularly regarding the risk of developing skin cancer. Chronic inflammation and immune-modulating treatments may contribute to this risk, making it important to understand how different drug classes affect skin cancer development. This review examines available evidence on the risk of melanoma and non-melanoma skin cancer associated with these therapies. While tumor necrosis factor inhibitors and Janus kinase inhibitors show a slightly increased risk, other treatments, such as anti-integrin agents and interleukin inhibitors, appear to have lower risks. Understanding these differences can help guide safer treatment choices and highlight the importance of regular skin cancer screening. Further long-term research is needed to ensure the safest possible management of inflammatory bowel disease.

## 1. Introduction

The treatment landscape for inflammatory bowel disease (IBD) has dramatically expanded with the development of biologic therapies and small molecule drugs [1]. These agents, including tumor necrosis factor (TNF) inhibitors, anti-integrins, interleukin (IL)-12/23 inhibitors, selective IL-23 inhibitors, Janus kinase (JAK) inhibitors, and sphingosine-1-phosphate receptor (S1P R) modulators, have revolutionized disease management by providing targeted control of inflammation [2]. However, their long-term safety profiles, particularly regarding cancer risk, warrant careful consideration.

Cancer risk in IBD patients arises from both disease-related and treatment-related factors [3]. Chronic inflammation has been recognized as a driver of oncogenesis, contributing to increased risks of colorectal cancer (CRC) [4] and extraintestinal malignancies [5]. Furthermore, immunosuppressive therapies used to manage IBD, while effective in controlling disease activity, may impair immune surveillance mechanisms essential for tumor suppression [6,7].

**Figure 1:**
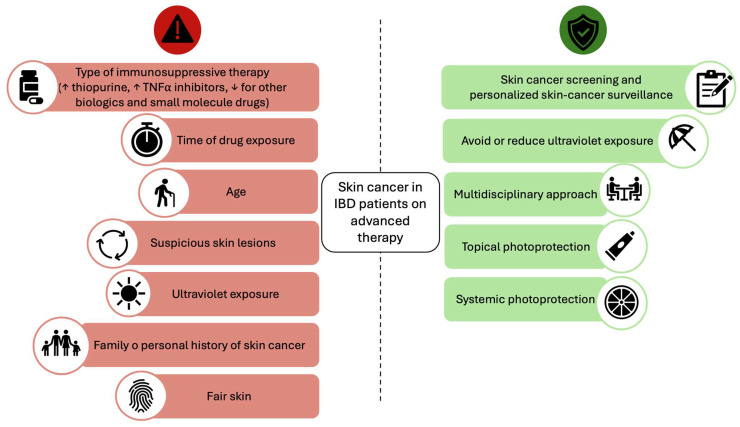
Factors contributing to skin cancer risk and strategies for mitigation. On the left side (red section), key risk factors are highlighted, including the type of immunosuppressive therapy (with increased risk associated with thiopurines and TNFα inhibitors, and potentially lower risk for other biologics and small-molecule drugs), duration of drug exposure, patient age, presence of suspicious skin lesions, ultraviolet (UV) exposure, family or personal history of skin cancer, and fair skin phenotype. On the right side (green section), recommended preventive strategies are outlined, emphasizing the importance of skin cancer screening and personalized surveillance, minimizing UV exposure, adopting a multidisciplinary approach, and utilizing both topical (sunscreen) and systemic photoprotection (nicotinamide, vitamins, minerals, polyphenols, carotenoids, and other antioxidants) to mitigate the risk of skin malignancies. This visual representation underscores the need for a tailored and proactive approach to skin cancer prevention in IBD patients receiving advanced therapy.

Within the spectrum of malignancies, particular attention has been directed toward skin cancers, which have emerged as a significant concern in this patient population. Skin cancer, including melanoma and non-melanoma skin cancer (NMSC), has emerged as a potential concern among patients treated with advanced immunomodulators. It is known that IBD patients have an increased risk of NMSC, with an overall annual incidence rate of 733 per 100,000 IBD patients (incidence rate ratio [IRR] 1.64; 95% CI 1.51–1.78) [5,8], which is particularly associated with the use of thiopurines [9]. Based on this evidence, the ECCO guidelines recommend regular skin cancer screening and the adoption of sun-protective measures for these patients [10] (Figure 1). Furthermore, a meta-analysis revealed a 37% increased risk of melanoma in patients with IBD, which appears to be independent of the use of biologic therapies [11].

TNF inhibitors, which were among the first biologics widely adopted for IBD, have been examined for increased risk of melanoma and NMSC [12], but studies have often been limited by confounding factors such as prior or concurrent thiopurine use and insufficient statistical power. Emerging biologics, such as IL-23 inhibitors, are under active investigation for their long-term safety profiles. Similarly, small molecule drugs, including JAK inhibitors and S1PR modulators, may carry unique immunological risks that necessitate further investigation. However, the risk of malignancy in IBD patients treated with these therapies varies depending on drug class, duration of therapy, and concomitant risk factors, such as a history of previous malignancy, ultraviolet exposure, or genetic predisposition [12].

This review aims to provide a comprehensive analysis of the current evidence regarding skin cancer risk in IBD patients treated with biologics and small molecule drugs. By exploring the underlying mechanisms, specific risks associated with each therapeutic class, and strategies for risk mitigation, this work seeks to inform clinical practice and guide patient management in the era of personalized medicine.

## 2. Materials and Methods

We conducted a comprehensive search of the Pubmed, Embase, and Scopus databases up to March 2025, with the aim of identifying studies regarding patients with IBD treated with advanced therapy and the risk of skin cancer. To achieve this, we employed specific search terms, such as ‘skin cancer’, ‘history of skin cancer’, ‘melanoma’, ‘non-melanoma skin cancer’ and ‘NMSC’, in conjunction with ‘Crohn’s disease’, ‘ulcerative colitis’, ‘CD’, ‘UC’, ‘inflammatory bowel disease’, and ‘IBD’. We limited our search to articles published in the English language.

Our screening process involved two independent reviewers (SB and FB) who initially assessed titles and abstracts to identify potentially relevant studies. Subsequently, we examined the full texts of these selected articles to determine their eligibility for inclusion. Additionally, we manually scrutinized the reference lists of these articles to ensure that no relevant studies were overlooked during the electronic search. The final inclusion of abstracts and articles was based on their relevance to our research objectives.

## 3. Results

### 3.1. TNFα Inhibitors

TNFα inhibitors are the most extensively studied biologic agents in the context of skin cancer risk. The cytokine TNFα serves as a key mediator of inflammation but exhibits a dual role, contributing to both tissue destruction and repair. In animal models, TNF plays a critical role in tumor cell destruction through its activation of natural killer cells and CD8 lymphocytes [13]. Additionally, TNF has been implicated in the selective destruction of tumor vasculature, while paradoxically also promoting tumor progression by facilitating tissue remodeling and stromal development required for tumor dissemination [14]. This duality of TNFα may help explain the anti-inflammatory efficacy of anti-TNF agents, alongside their potential association with an increased risk of skin cancer and other malignancies. Numerous studies have explored their potential cancer risks. The findings indicate that TNFα inhibitors do not increase the overall risk of malignancy in IBD patients [10]. However, challenges remain in accounting for confounding factors, such as concurrent thiopurine use, disease severity, and patient demographics, leaving uncertainties about the risks associated with certain cancer sub-types.

A 2012 study utilizing a health insurance claims database analyzed data from 10,879 IBD patients, each matched with four non-IBD controls. The findings indicated that biologic therapy, including TNFα inhibitors and natalizumab, was significantly associated with an increased risk of melanoma in the IBD population (OR: 1.88; 95% CI: 1.08–3.29). This association was particularly notable in patients with CD (OR: 1.94; 95% CI: 1.03–3.68), but not in those with UC (OR: 1.73; 95% CI: 0.53–5.63) (Table 1) [15].

This finding has not been consistently replicated in subsequent studies. For instance, a Danish population registry study reported no significant association between TNFα inhibitors exposure and melanoma (relative risk [RR]: 1.31; 95% CI: 0.63–2.74) [16]. Additionally, a 2020 systematic review and meta-analysis, which included 7,901 IBD patients treated with TNFα inhibitors and 135,370 biologic-naive patients, found no statistically significant link between TNFα inhibitors therapy and melanoma risk in IBD patients (pooled relative risk [pRR]: 1.20; 95% CI: 0.60–2.40) [17]. Similar results were observed in the same meta-analysis for patients with rheumatoid arthritis (RA) receiving biologics (pRR: 1.20; 95% CI: 0.83–1.74) and for patients with psoriasis (HR: 1.57; 95% CI: 0.61–4.09) compared to those on conventional therapies [17].

Several studies have found no association between NMSC and the use of TNFα inhibitors in IBD patients. A large retrospective analysis of a US claims database reported no significant link between TNFα inhibitor therapy and NMSC in IBD patients (OR: 1.14; 95% CI: 0.95–1.36), with similar results observed when analyses were limited to patients with CD (OR: 1.16; 95% CI: 0.95–1.41) or UC (OR: 1.06; 95% CI: 0.69–1.64) [15]. Furthermore, a 2014 pooled analysis of clinical trials evaluating adalimumab in CD (3050 patient-years (PY) of exposure) found no association between adalimumab monotherapy and NMSC (standardised incidence ratio [SIR]: 1.20; 95% CI: 0.39–2.80) [18]. On the other hand, a recent systematic review of 28 studies, including 298,717 IBD patients, identified 692 malignancies (1%) in patients exposed to TNFα inhibitors. Among these, NMSC was the most frequently reported malignancy (123/692; 17.8%) [19], occurring at rates comparable to those expected in the general non-IBD population [16].

Current evidence does not suggest an additional increase in the risk of skin cancers in IBD patients treated with combination therapy involving TNFα inhibitors and a thiopurine or methotrexate when compared to the cancer risks associated with monotherapy using either agent.

An initial pooled analysis of clinical trial data for adalimumab in CD suggested that combination therapy with a thiopurine or methotrexate was associated with an increased risk of both NMSC (RR: 3.46; 95% CI: 1.08–11.06) and other malignancies (RR: 2.82; 95% CI: 1.07–7.44) compared to adalimumab monotherapy [18]. However, this finding was not corroborated by subsequent data from the PYRAMID and TREAT post-marketing registries (for adalimumab and infliximab, respectively), which reported no statistically significant differences in exposure-adjusted malignancy rates—excluding lymphoma—between CD patients receiving TNFα inhibitors with or without concomitant thiopurine therapy at baseline [20,21]. Furthermore, case-control studies did not identify concomitant immunosuppressive therapy as a risk factor for malignancy development, with controls consisting of IBD patients who had never been treated with TNFα inhibitors, thiopurines, or methotrexate [16,20,22,23].

Overall, while initial studies raised concerns about an elevated risk of skin cancer associated with TNFα inhibitors, more recent and comprehensive analyses suggest that these agents, whether used as monotherapy or in combination with immunosuppressants, do not significantly increase the incidence of melanoma or NMSC in patients with IBD.

**Table 1:** Summary of studies of advanced therapy in inflammatory bowel disease with incidence of melanoma and non-melanoma skin cancer.

Author	Therapy	Type of Study	Subjects	Melanoma	NMSC	Increased Risk
Long MD et al. [15]	TNFα inhibitors and natalizumab	Retrospective	10,879 IBD patients	OR: 1.88 (95% CI: 1.08–3.29)	OR: 1.14 (95% CI: 0.95–1.36)	Yes, only for melanoma
Nyboe Andersen N et al. [16]	TNFα inhibitors	Retrospective	56,146 IBD patients	RR: 1.31 (95% CI: 0.63–2.74)	-	No
Esse S et al. [17]	TNFα inhibitors	Systematic review and meta-analysis	7901 IBD patients	pRR: 1.20 (95% CI: 0.60–2.40)		No
Osterman MT et al. [18]	Adalimumab Adalimumab + thiopurine or methotrexate	Comparative study	1594 CD patients	-	SIR: 1.20 (95% CI: 0.39–2.80) RR: 3.46 (95% CI: 1.08–11.06	No Yes
Muller M et al. [19]	TNFα inhibitors	Systematic review	298,717 IBD patients	-	123/692, 17.8%	No
Lichtenstein GR et al. [20]	Infliximab	Prospective	6273 CD patients	SIR: 1.05 (95% CI 0.42, 2.16)	0.16 events/100 PY	No
DʼHaens G et al. [21]	Adalimumab	Prospective	5025 CD patients	<0.1 events/100 PY	0.3 events/100 PY	No
Cohen RD et al. [24]	Vedolizumab	Retrospective	32,752 IBD patients	12 cases of skin malignancies		No
Ghosh S et al. [25]	Ustekinumab	Pooled Safety Analysis of clinical trials	2575 IBD patients	0.11 events/ 100 PYs	0.38 events/100 PY (95% CI: 0.22–0.59)	No
Ferrante M et al. [26]	Risankizumab	RCT	1147 CD patients	-	0.6 events/100 PY	No
Sands BE et al. [27]	Mirikizumab	RCT	1162 UC patients	-	-	No
Rubin DT et al. [28]	Guselkumab	RCT	701 UC patients	-	-	No
Olivera PA et al. [29]	Tofacitinib, filgotinib, baricitinib, and upadacitinib	Meta-analysis	5987 IBD patients		RR: 1.21 (95% CI: 0.19–7.65)	No
Panés J et al. [30]	Tofacitinib	RCT	1157 UC patients	2 cases of melanoma	IR: 0.71/100 PY(95% CI, 0.45–1.07).	No
Russell MD et al. [31]	Tofacitinib, baricitinib, upadacitinib, filgotinib, peficitinib	Meta-analysis	82,366 PY of exposure (RA, PsA, PsO, axSpA, IBD or AD)	-	IRR: 1.93 (95% CI 1.19–3.12)	Yes
Siegel C et al. [32]	Ozanimod	RCT	823 UC patients	-	EAIR: 0.1/100 PY OLEw94 EAIR: 0.04/100 PY OLEw142	No
Vermeire S et al. [33]	Etrasimod	RCT	956 UC patients	-	-	No

RCT: randomized controlled trial, CD: Crohn’s disease, UC: ulcerative colites, IBD: inflammatory bowel disease, RA: Rheumatoid Arthritis, PsA: Psoriatic Arthritis, PsO: Psoriasis, axSpA: Axial Spondyloarthritis, AD: Atopic Dermatitis, OR: odds ratio, RR: relative risk, PY: patient-years, CI: confidence interval, pRR: pooled relative risk, SIR: standardised incidence ratio, IRR: incidence rate ratios, IR: incidence rate.

### 3.2. Vedolizumab

Vedolizumab is a monoclonal antibody that targets the α4β7 integrin with a favorable benefit-risk profile [24].

Post-marketing surveillance data from the Vedolizumab Global Safety Database, with over four years of follow-up, have provided medium-term safety information for patients with CD or UC receiving vedolizumab treatment. These data, representing 208,050 PY of vedolizumab exposure (calculated based on an assumed 8-week dosing interval), did not indicate an overall increased risk of malignancy in either CD or UC [24]. A total of 251 malignancies were reported among patients with CD or UC (134 and 117 cases, respectively), accounting for less than 1% of all adverse events. In CD patients, 12 cases of skin malignancies were recorded (12 cases, unspecified and other). While in UC patients, no skin cancer occurred.

These findings support the reassuring safety profile of vedolizumab, suggesting no significant increase in the risk of overall malignancy or skin cancer in patients with CD or UC during medium-term follow-up.

### 3.3. Ustekinumab

Ustekinumab, an inhibitor targeting the shared p40 subunit of IL-12 and IL-23, has shown a favorable safety profile regarding skin cancer.

Safety data were presented in a pooled safety analysis through 5 years in CD and 4 years in UC. A total of 2575 patients were treated with 4826 PY, with follow-up showing no increase in skin cancer risk [25]. The NMSC rates were 0.32 per 100 PYs (95% CI: 0.07–0.93) in the placebo group and 0.38 per 100 PYs (95% CI: 0.22–0.59) in the ustekinumab group. The most frequently reported malignancies (>2 patients) were basal cell carcinoma (placebo: 0.14 [2 patients] vs. ustekinumab: 0.54 [14 patients]), squamous cell carcinoma (placebo: 0.14 [2 patients] vs. ustekinumab: 0.23 [6 patients]), and melanoma (placebo: 0.07 [1 patient] vs. ustekinumab: 0.11 [3 patients]). Observational studies align with these findings, indicating that malignancies were rare [34,35]. In a multicenter cohort study of 142 CD patients, dose escalation of ustekinumab to every 4 weeks did not lead to an increased risk of adverse events, including malignancies. Over a follow-up period of up to 52 weeks, only one case of NMSC was reported [36].

Overall, current evidence indicates that ustekinumab maintains a favorable safety profile with a low incidence of skin cancer in patients with IBD, even with prolonged exposure and dose escalation.

### 3.4. Selective IL-23 Inhibitors (e.g., Risankizumab, Mirikizumab, Guselkumab)

Risankizumab (approved for CD), mirikizumab (approved for UC), and guselkumab are drugs that selectively inhibit the IL-23 pathway by targeting its p19 subunit. Given the relatively recent introduction of this therapy in IBD, real-world safety data regarding melanoma and NMSC are limited, but clinical trials report a reassuring safety profile.

Long-term safety data for risankizumab in CD have been evaluated in the FORTIFY Open-Label Long-term Extension study (3 years) [26], which included 1147 patients who had completed the 52-week maintenance phase in the FORTIFY substudies [37], or the phase 2 open-label extension [38]. These analyses revealed no new safety concerns related to malignancies. Notably, in the FORTIFY study, there was only one case of NMSC reported in the withdrawal group (patients who transitioned from risankizumab to subcutaneous placebo). This case accounted for 1% of the withdrawal group (1/184) and corresponded to an incidence rate of 0.6 events per 100 person-years, with a total exposure of 160.4 person-years.

Considering mirikizumab, long-term safety data in UC have been evaluated in LUCENT-3 Open-Label Extension Study (2 years) [27]. During 104 weeks of continuous treatment, no malignancies were observed.

Guselkumab has shown its efficacy in UC and CD in phase 2 and 3 trials (QUASAR [28], GALAXI series [39] and GRAVITI [40]), and it has been approved by Food and Drug Administration for UC [41] but not yet by EMA. However, only in the QUASAR study, two cases of NMSC were recorded in the guselkumab group during the induction phase (0.5%) and two cases of NMSC in the placebo group during the maintenance phase (1%).

Nevertheless, given the relatively recent introduction of these therapies, ongoing pharmacovigilance remains critical to fully assess its long-term safety profile regarding melanoma and other malignancies.

### 3.5. JAK Inhibitors (e.g., Tofacitinib, Filgotinib and Upadacitinib)

Janus kinase (JAK) inhibitors modulate multiple cytokine pathways and have raised concerns about their impact on malignancy risk. Tofacitinib, a JAK inhibitor primarily targeting JAK1 and JAK3, was approved for the treatment of UC. Instead, upadacitinib (approved for both UC and CD) and filgotinib (approved for UC) are selective JAK 1 inhibitors.

Evidence from the IBD population is satisfactory. A meta-analysis of randomized controlled trials, which included 5987 patients with IBD, found no significant differences in the risk of NMSC associated with JAK inhibitors compared to placebo or active comparators (RR: 1.21; 95% CI: 0.19–7.65) [29]. Focusing on tofacitinib in UC, available safety data from the completed global clinical development program encompass up to 9.2 years of drug exposure [30]. Particularly, a total of 1157 patients received at least one dose of tofacitinib at either 5 mg or 10 mg twice daily, with a total drug exposure of 3202.0 PY. There were only two cases of melanoma, while NMSCs were reported in 23 patients, with an incidence rate (IR) of 0.71 per 100 PY (95% CI, 0.45–1.07). Among these cases, six occurred in patients receiving the predominant dose (PD) of tofacitinib 5 mg twice daily (IR 0.66, 95% CI 0.24–1.43), while 17 were observed in the PD tofacitinib 10 mg twice daily group (IR 0.73, 95% CI 0.43–1.17). Specifically, 17 patients were diagnosed with BCC, and 11 with SCC.

On the other hand, a systematic review with meta-analysis of 62 randomized controlled trials (RCTs) and 16 long-term extension (LTE) evaluated the safety of JAK inhibitors compared to placebo, TNFα inhibitors, and methotrexate in adults with various immune-mediated conditions, such as rheumatoid arthritis, psoriatic arthritis, psoriasis, and IBD [31]. Compared to placebo (IRR 0.71; 95% CI 0.44–1.15) and methotrexate (IRR 0.77; 95% CI 0.35–1.68), JAK inhibitors showed no significant increase in malignancy risk, including NMSC. However, compared to TNFα inhibitors, JAK inhibitors were associated with a higher incidence of malignancies (IRR 1.50; 95% CI 1.16–1.94); in particular, the incidence of NMSC was significantly higher with JAK inhibitors compared to TNFα inhibitors (IRR 1.93; 95% CI 1.19–3.12).

To gain more comprehensive insights into the safety profile of other JAK inhibitors, such as upadacitinib and filgotinib, longer observational periods and an increased volume of real-world data are needed.

### 3.6. S1P Receptor Modulators

Sphingosine-1-phosphate (S1P) receptor modulators, such as ozanimod and etrasimod, are newer agents with limited long-term data. Clinical trial data consistently support the favorable safety profile of these drugs, demonstrating no significant skin cancer risks over the duration of the studies.

In particular, an interim analysis from the True North open-label extension (OLE) study evaluates safety data from 823 UC patients (total exposure of 2536 PY), following approximately five years of continuous ozanimod treatment. [42]. BCC was reported in one patient (0.1%) in the OLE cohort in week 94, with an exposure-adjusted incidence rate (EAIR) of 0.1 per 100 PY, and in one patient (0.1%) in the OLE cohort in week 142, with an EAIR of 0.04 per 100 PY [32]. Regarding etrasimod, safety data analyzed from pooled global clinical trials with up to 2.5 years of follow-up (956 UC patients received ≥1 dose of etrasimod with 769.3 PY exposure) reported no cases of skin cancer [33].

In conclusion, clinical trial data to date do not indicate a substantial increase in NMSC and melanoma risk. However, the mechanism of action, which involves lymphocyte trafficking inhibition, warrants cautious monitoring.

Moreover, given the relatively short duration of post-marketing experience, continued evaluation is necessary.

A summary of all classes of drugs used as advanced therapy in IBD and their association with the risk of melanoma and NMSC is available in Table 2.

## 4. Discussion

The risk of skin cancer in patients with IBD undergoing advanced therapies is a multifaceted issue that necessitates a personalized approach to screening and surveillance [12]. This review provides a comprehensive summary of the available evidence on the risk of skin cancer associated with the use of advanced therapies in patients with IBD. Although definitions of IBD may vary across studies, the included populations consistently comprised patients with moderate-to-severe disease requiring advanced therapies, which supports the comparability of safety outcomes despite potential variability in diagnostic criteria.

Understanding that the risk profile varies between individuals and therapeutic regimens is critical for optimizing patient outcomes.

Skin cancer risk is influenced by various factors, including patient age, immune status [43], and prior exposure to immunosuppressive agents [5,43]. However, the current literature lacks sufficient data to allow a detailed analysis of how these individual risk factors interact with specific advanced therapies.

Particularly, elderly IBD patients, over 65 years, have an increased risk of developing cancer [44], including skin cancer due to cumulative UV exposure and immunosenescence. Among other factors besides age, it is essential to consider a personal or family history of skin cancer, sun exposure, genetic predisposition, fair skin and the presence of suspicious skin lesions, such as actinic keratosis or dysplastic nevi [45,46]. These patients require therapies with a skin safety profile while also benefiting from a tailored screening approach, incorporating intensified surveillance based on individual risk factors. An important subgroup to consider includes patients with a history of skin cancer and IBD. In particular, some small and generally underpowered studies have shown that TNFα inhibitors are not associated with worse tumor progression, recurrence, or survival in melanoma and NMSC [47,48,49].

Given the complexity and variability of individual risk profiles, appropriate monitoring strategies are critical to ensure the timely identification and management of skin lesions. However, guidelines generally recommend a dermatologic evaluation before initiating treatment, but they do not specify a standardized frequency for follow-up dermatologic visits during therapy [10]. Currently, dermatological screening stratified by drug type is not feasible due to insufficient data. Therefore, the decision regarding the timing of dermatologic assessments should be made in collaboration with the dermatologist, considering the clinical characteristics and individual risk factors of each patient. Implementing personalized surveillance protocols ensures that early detection strategies are applied judiciously, balancing efficacy with resource allocation.

In parallel, a deeper understanding of the biological mechanisms underlying skin cancer development in patients receiving advanced therapies is crucial to inform both risk stratification and preventive strategies. The underlying mechanisms contributing to skin cancer risk in patients treated with advanced therapies remain under investigation. For TNFα inhibitors, the disruption of tumor necrosis factor signaling may impair immune surveillance against early neoplastic changes. JAK inhibitors, on the other hand, can affect multiple cytokine pathways that are critical for tumor immunity [3]. Additionally, IL-23 inhibition may enhance antitumor responses by targeting the IL-23/IL-23 receptor axis, leading to the destabilization of regulatory T cells (Tregs) within the tumor microenvironment. IL-23 is a cytokine that promotes regulatory T cell proliferation and IL-10 expression, both of which can suppress immune-mediated tumor cell killing. Therefore, inhibiting IL-23 could potentially reduce the immunosuppressive effects of Tregs and improve the efficacy of existing cancer therapies [50]. Understanding these mechanisms offers opportunities to develop targeted preventive interventions.

Nowadays, prevention strategies go beyond simply avoiding or reducing UV exposure—it is important to take proactive steps to shield the skin. This can be achieved through two main strategies: topical and systemic photoprotection [51]. Topical photoprotection relies on sunscreens applied directly to the skin, which are essential but not without limitations. Their effectiveness is often short-lived, proper application can be inconsistent, and they provide minimal systemic protection [51]. Additionally, while rare, some individuals may experience contact dermatitis from sunscreen ingredients [51]. On the other hand, systemic photoprotection offers a promising alternative. This approach involves taking oral compounds with photoprotective and anti-photocarcinogenic properties, such as nicotinamide, vitamins, minerals, polyphenols, carotenoids, and other antioxidants [52]. These substances work by enhancing the body’s natural defense against UV damage, helping to prevent skin cancer and photoaging. Their protective effects stem from a variety of mechanisms, including antioxidant, anti-inflammatory, and immunomodulatory actions, making them a valuable addition to comprehensive sun protection strategies [53]. However, data regarding these strategies in the setting of IBD patients treated with advanced therapy are not yet available.

For established therapies, such as TNFα inhibitors, long-term safety data provide a well-defined risk profile. Studies consistently indicate that while TNFα inhibitors are associated with an increased risk of melanoma (OR: 1.88; 95% CI: 1.08–3.29) [15], the risk is manageable with appropriate monitoring. As recommended by guidelines, skin-cancer surveillance and sun protection measures tailored to individual risk should continue to be emphasized [10]. In contrast, newer agents lack extensive real-world data and observational studies. Real-world evidence will be essential to bridging this knowledge gap and refining clinical practice guidelines.

As treatment strategies evolve, particularly with earlier and more intensive use of advanced therapies, it becomes increasingly important to consider how cumulative exposure may influence long-term skin cancer risk. The top-down approach, often involving early use of advanced therapies, could contribute to a higher cumulative risk of skin cancer when multiple agents are used in combination [53]. Nevertheless, the appropriate use of skin cancer prevention strategies, such as patient education on UV protection and a multidisciplinary approach with dermatologists, could help counterbalance the theoretical risks associated with combination therapy. Interestingly, evidence suggests that combining TNFα inhibitors with thiopurines, such as azathioprine, does not significantly increase the risk of skin cancer beyond what is observed with thiopurine monotherapy [20,21]. This finding underscores the importance of assessing individual drug contributions to overall risk and highlights the potential for dual therapy, combining more advanced therapy, to be safely utilized in specific patient populations under appropriate monitoring.

## 5. Conclusions

The evolving landscape of advanced therapies for IBD underscores the need for a nuanced understanding of skin cancer risk. Apart from TNFα inhibitors, which may increase the risk of melanoma, other advanced therapies currently appear to have a reassuring safety profile regarding skin cancer development. Clinicians should incorporate risk stratification tools, engage dermatology early in the management process, and actively educate patients on effective sun protection measures. However, continued research into mechanistic pathways, and more studies assessing real-world safety data and combination therapy risks, will further refine our approach to balancing therapeutic efficacy with long-term safety in IBD management.

## Figures and Tables

**Table 2:** Skin cancer risk with advanced therapy in inflammatory bowel disease.

	Melanoma	NMSC
TNFα inhibitor	Yes	No
Ustekinumab	No	No
Vedolizumab	No	No
Selective IL-23 inhibitors	Unknown *	Unknown *
JAK inhibitors	No **	No **
S1P modulators	Unknown *	Unknown *

* Only clinical trial data available. ** Long-term data only for tofacitinib.
